# Primary Extraosseous Central Nervous System Ewing Sarcoma in Children: A Rare Case Series from a Major Public Neuro-Oncology Center in Pakistan

**DOI:** 10.12669/pjms.41.13(PINS-NNOS).13489

**Published:** 2025-12

**Authors:** Rahat Ul Ain, Sundas Irshad, Laeeq ur Rehman, Rabia Qaiser, Mahvish Hussain, Mahwish Faizan

**Affiliations:** 1 Rahat Ul Ain, MBBS, FCPS, FCPS, FPNO, Department of Pediatric Hematology/Oncology, The Children’s Hospital Lahore, Lahore, Pakistan; 2 Sundas Irshad, MBBS, Punjab Institute of Neuro Sciences,Lahore, Pakistan; 3 Laeeq ur Rehman, MBBS, FCPS, Department of Pediatric Neurosurgery, The Children’s Hospital Lahore, Lahore, Pakistan; 4 Rabia Qaiser, MBBS, FCPS, Department of Pediatric Radiology, The Children’s Hospital Lahore, Lahore, Pakistan; 5 Mahvish Hussain, MBBS, FCPS, Department of Pediatric Pathology, The Children’s Hospital Lahore, Lahore, Pakistan; 6 Mahwish Faizan, MBBS, MCPS, FCPS, FPHO, Department of Pediatric Hematology/Oncology, The Children’s Hospital Lahore, Lahore, Pakistan

**Keywords:** Ewing Sarcoma, Neoplasms, Connective and Soft Tissue, Brain Neoplasms, Central Nervous System Neoplasms, Child

## Abstract

Primary extraosseous central nervous system Ewing Sarcoma (CNS-EES) in children is a rare disorder, and this study aimed to document the experience of dealing with these rare pathologies of CNS in our center. This ambidirectional descriptive case series included all consecutive cases of CNS-EES in children under the age of 16 years presenting to the The Children’s Hospital Lahore, Pakistan, from January 2023 to December 2024. During the 24-month study period, 4 out of 139 incident CNS tumor cases at our center were diagnosed as primary CNS-EES, corresponding to a proportional incidence of 2.9% (95% CI: 1.1-7.2%). The median age of presentation was 8.5 years, with equal gender distribution. Pain was the first presenting symptom, with a median symptom duration of 5.75 weeks. Half of the cases presented with a cerebral hemispheric mass and half with a spinal mass. The median event-free survival was 4.5 weeks with a survival rate of 25%. All cases were diagnosed based on a round blue cell morphology with a positive NKX2.2. Primary CNS-EES is not very infrequent in our population, with possible parietal lobe predilection and association with cancer predisposition syndrome. In resource-limited settings, NKX2.2 might serve as a fair substitute to diagnose CNS-EES.

## INTRODUCTION

Ewing sarcoma (ES) is known to be the second most common malignant tumor of the bones in children and adolescents, but can also present with extra-osseous/soft tissue tumors.^1^ Primary extraosseous ES arising from the central nervous system (CNS-EES) is a rare entity,^2^ and is rarer in the pediatric population.^3^ ES has been recently redefined by the World Health Organization (WHO) as soft tissue and bone tumors with a specific combination of gene fusion between EWSR1/FUS and the genes encoding the ETS (E26 transformation-specific) family of transcription factors.^4^ However, for resource-limited settings, classical histopathology and immunohistochemistry are still the gold standard for diagnosis. Even in institutes having molecular testing, a round blue cell sarcoma morphology and positivity of CD99, NKX2.2, and PAX7 are commonly used either for rapid diagnosis of ES or in cases where molecular testing is inconclusive.^5^,^6^

Primary CNS-EES is treated in a similar way as osseous or extraosseous ES elsewhere in the body, with local maximum safe surgical excision followed by adjuvant therapy in the form of radiotherapy and chemotherapy.^1^,^2^ The survival outcomes of primary CNS-EES are also comparable to the osseous and extraosseous ES,^7^ but pediatric cases of CNS-EES have not been exclusively studied due to the rarity of this group. This study aimed to present the experience of pediatric CNS-EES at a single institute in a resource-limited country.

## METHODOLOGY

This ambidirectional descriptive case series included all consecutive cases of CNS-EES in children under the age of 16 years presenting in the Department of Pediatric Hematology/Oncology and the Department of Pediatric Neurosurgery at the University of Child Health Sciences, The Children’s Hospital Lahore, Pakistan. The data were collected from the hospital records retrospectively from January 2023 and prospectively from March 2024 to December 2024. The institutional ethical committee approval was taken before data collection (no. 803/CH-UHS, dated 05-03-2024). The study included all the children diagnosed with Primary CNS-EES under the age of 16 years. The clinical data, including age, gender, presenting complaints, symptom duration, tumor location, radiological features, treatment, and outcome, were collected in a case report form. All cases were followed till February 2025 for survival analysis. Event-free survival (EFS) was defined as the interval between time to diagnosis till either the death of the patient, treatment abandonment, or the data cut-off point. Proportional incidence was determined as the number of cases of the specific tumor pathology divided by the total number of incident solid tumors over 24 months, with 95% confidence intervals calculated using Wilson’s method.

## RESULTS

**Fig.1:**
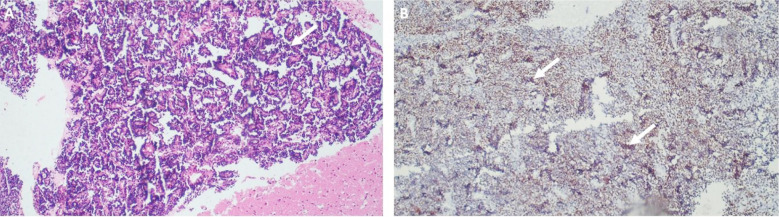
A. Photomicrograph of H & E staining in Ewing’s sarcoma in brain with nuclear palisading x 20. B. Photomicrograph of positive nuclear staining of NKX2.2 in Ewing Sarcoma x 20

During the 24-month study period, four out of 139 incident CNS tumor cases at our center were diagnosed as primary CNS-EES, corresponding to a proportional incidence of 2.9% (95% CI: 1.1-7.2%). The median age of presentation was 8.5 years (8-13 years), with a male-to-female ratio of 1:1. The median symptom duration was 5.75 weeks (1-8 weeks). The histopathological diagnosis of ES was made on the basis of a radiologically primary extraosseous CNS tumor with histopathological findings of round blue cell morphology with positive NKX2.2 immunohistochemical marker (Fig.1) and a multidisciplinary tumor board (MDT) consensus. The staging workup included complete neuro-axis imaging (MRI brain with contrast and MRI whole spine with contrast), CT neck, chest, abdomen, and pelvis with IV contrast, bone scan, and bilateral bone marrow biopsy.

**Fig.2:**
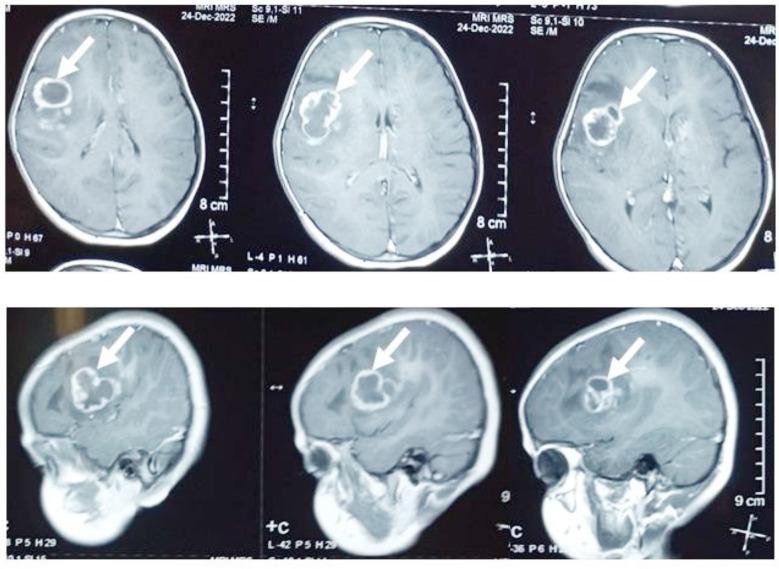
Case 1: MRI brain T1W post-contrast: Multiple marginally-enhancing and nodular lesions in right frontoparietal lobe with extensive peri-lesional vasogenic edema, ill-defined, heterogenous enhancement of left caudate nucleus head, and multifocal bilateral intra-axial lesion showing FLAIR and T2 hyperintensity.

The intracerebral lesions shared the common radiological features of parietal lobe involvement and marginally enhancing, solid/cystic lesions (Fig.2 & 4), but the cases with spinal lesions did not show any radiological similarity with each other. Two of the cases (one cerebral and one spinal) could receive adjuvant therapy post-surgery (chemotherapy/radiotherapy), while only one achieved radiological remission after adjuvant chemo/radiotherapy and survived at the study data cut-off point. The response assessment was done with post-contrast MRI of the primary site/s of the disease in an MDT. The median event-free survival was 4.5 weeks (4-40 weeks) (Table-I).

**Fig.3:**
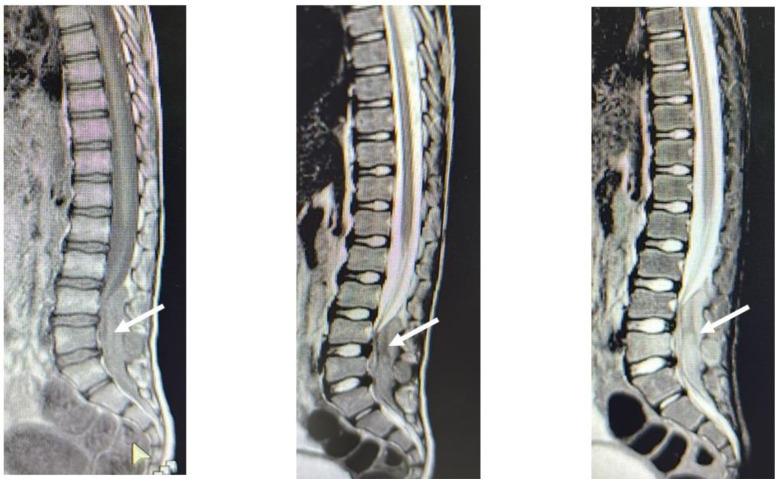
Case 2: MRI lumbo-sacral spine: A 7.8 x 1.6 cm enhancing soft tissue intra-spinal mass from the upper border of L4 up to S2 level with extension through neural foramina into para-spinal regions.

**Fig.4:**
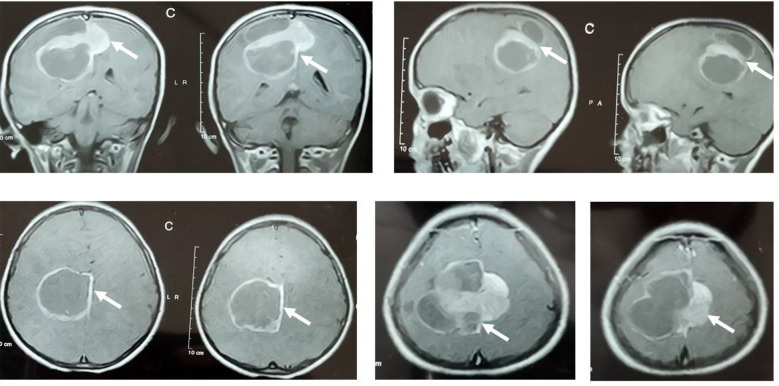
Case 3: MRI brain T1W post-contrast images: multi-lobulated heterogeneously enhancing, 5.5*6.7*6.3cm lobulated solid/cystic mass lesion in the right parafalcine region along the parietal lobe crossing midline and significantly compressing ipsilateral lateral ventricle and distal half of body of corpus callosum.

**Table-I:** Clinical characteristics of extra-osseous CNS Ewing sarcoma in children.

Case No.	Age (yr)	Sex	Primary site	CNS findings	Neuro-cutaneous stigmata	Post-op status	Metastasis	EFS	Outcome	Suspected Cancer predisposition syndrome
1	8	F	Right parieto-temporal lobe	NAD	Café au lait macules	Gross residual	None	5 weeks	Expired before adjuvant treatment	Turcot Syndrome
2	9	M	Spinal (L4-S2) Intraspinal, extradural	Paraplegia	None	Not known	None	4 weeks	Lost to follow up after surgery	None
3	8	F	Right parietal lobe	Left hemiplegia	Café au lait macules	Gross residual	None	40 weeks	On chemo-therapy (complete remission)	Constitutional Mismatch Repair Deficiency
4	13	M	Spinal (cervical) Intramedullary, Intradural	Quadriparesis	None	Gross residual	Present	4 weeks	Expired	None

### CASE-1:

An eight-year-old girl, a resident of Lahore (Punjab, Pakistan), presented in January 2023 with complaints of headaches and vomiting for one week. She was a product of a consanguineous marriage, and her younger sister died of a brain tumor; therefore, she was diagnosed within a week of the development of her symptoms. On examination, she had a normal physical exam except for having multiple café au lait macules over her body. The Magnetic Resonance Imaging (MRI), Brain with contrast, showed a mass in the right fronto-parietal lobe, suspected pediatric high-grade glioma (Fig.2). After discussion in the multidisciplinary tumor board, maximum-safe surgical resection was done. The histopathology report showed a neoplasm comprising atypical cells with hyperchromatic nuclei and an increased N/C ratio, arranged in sheaths, with areas of necrosis.

Morphological features favored a high-grade neoplasm, likely a round blue cell tumor. Positive NKX2.2, INI-1 retained, high Ki67, but negative Synaptophysin, CD99, GFAP, LCA, and Desmin. This suggested the possibility of Ewing Sarcoma. Further translocation testing was not available. Soon after discharge, she presented in the emergency room with intestinal obstruction and per-rectal bleeding. Laparoscopic reduction of intussusception was done with visualization of multiple polyps in the colon, but she unfortunately succumbed to hemorrhagic shock. Clinical suspicion of Turcot syndrome was made due to a history of a brain tumor in an elder sibling, positive consanguinity, café au lait macules, and multiple polyps in the colon.

### CASE-2:

A nine-year-old boy, a resident of Shakargarh (Punjab, Pakistan), presented in January 2024 with backache for two months, followed by pain in his legs, progressive difficulty in walking, and loss of bladder/bowel control. There was no consanguinity or family history of cancers. On examination, he had paraplegia with a power of 0/5 in both lower limbs. The MRI of the lumbosacral spine with contrast showed an intraspinal, extradural soft tissue mass from L4 to S2 (Fig.3). Surgical excision was done, and the histopathology report showed a malignant neoplasm composed of small round to oval cells that were arranged in sheets and nests with scant cytoplasm, hyperchromatic nuclei, and an increased N/C ratio. In addition, necrosis and bony trabeculae with nervous tissue were also seen. The histopathological features were of a round blue cell tumor with positive NKX2.2, 50% Ki67, and negative CD20 and CD3, suggestive of Ewing Sarcoma. Translocation testing was not available. The patient was lost to follow-up after the surgery.

### CASE-3:

An eight-year-old girl, resident of Gilgit (Gilgit-Baltistan, Pakistan), presented in May 2024 with complaints of headaches for two months, followed by left leg and arm weakness for 1.5 months. She was a product of a consanguineous marriage with an insignificant family history. On examination, she had multiple hyperpigmented macules over the nasal bridge and cheeks, and a power of 1/5 on the left side. The MRI brain with contrast showed a solid cystic mass in the right parietal lobe (Fig.4). She underwent subtotal resection and the histopathology report showed sheets of round blue cell tumor cells with scant cytoplasm and hyperchromatic nuclei, positive immunohistochemical (IHC) stains of CD99, NKX2.2, BCL2, a high Ki67, and negative IHC stains of Cytokeratin AE1/AE3, LCA, SALL-4, Desmin, CD20, CD10, suggestive of Ewing Sarcoma.

The case was discussed in the multidisciplinary tumor board and was recommended to be treated as a primary CNS-EES with EuroEwing99 protocol. After four cycles of chemotherapy and radiation therapy (54Gy/30Fr), the repeat imaging showed no residual disease. Her hemiplegia settled, and the power resumed to 5/5. She is currently completing her adjuvant chemotherapy with the disease in complete remission. No major chemo/radiotherapy toxicity observed during the adjuvant treatment. Clinical suspicion of Constitutional Mismatch Repair Deficiency Syndrome was made due to positive consanguinity, multiple café au lait macules, and a high-grade brain tumor pathology

### CASE-4:

**Fig.5:**
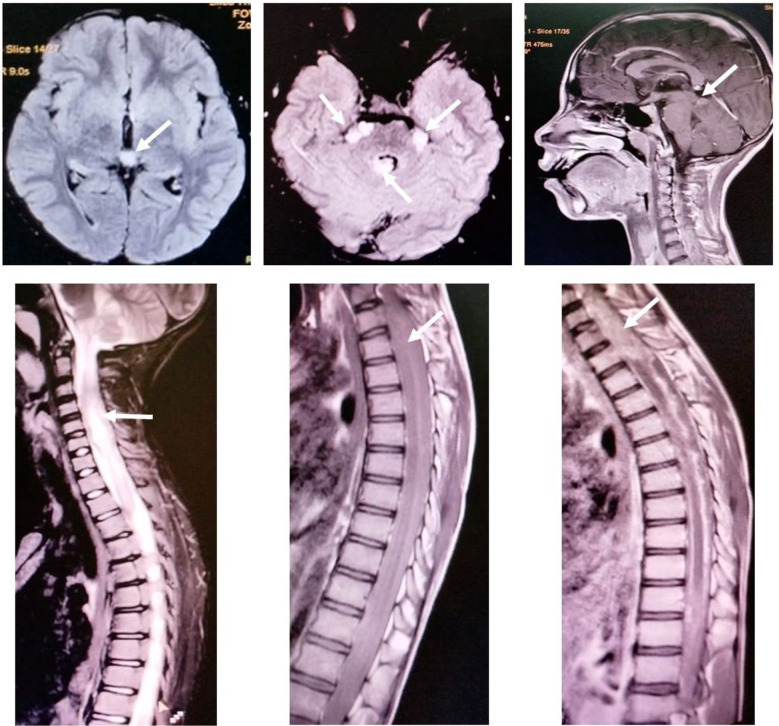
Case 4: MRI Brain and cervical spine with contrast: A 7.7 x 1.4 cm long segment heterogeneous enhancing lesion in the cervicodorsal cord. Multifocal heterogeneous intensity minimally enhancing intracranial extra-axial lesions in the posterior cranial fossa, interpeduncular and quadrigeminal cistern.

A thirteen-year-old boy, resident of Khanewal (Punjab, Pakistan), presented in December 2024 with complaints of pain in the right shoulder, palpitations, and dry mouth for 25 days, right-sided body weakness and pain in the back of the neck for two weeks, left-sided body weakness, vomiting, and constipation for one week. He was a product of a consanguineous marriage with an insignificant family history. On examination, no cutaneous stigmata of cancer predisposition/neuro-cutaneous syndrome, LPS of 0, respiratory distress, loss of bladder and bowel control, and quadriparesis with a power of 2/5 on the left side and 4/5 on the right. The MRI cervical spine and brain with contrast showed a cervical intramedullary spinal mass with multifocal intracranial extra-axial lesions in the posterior cranial fossa (Fig.5). He underwent subtotal resection of the spinal lesion, and histopathology showed a neuronal pattern with nests of spindle cell proliferation. In addition, small hyperchromatic cells with scant cytoplasm, calcifications, and delicate vessels but no mitosis or endothelial proliferation. Immunohistochemistry showed positive NKX2.2, and S100, high Ki67, and negative Synaptophysin. The morphology and immunohistochemistry favored Ewing Sarcoma. After discussion in the multidisciplinary tumor board, chemotherapy (EuroEwing99 protocol) was started, but due to the progressive diaphragmatic paralysis, he succumbed to ventilator-associated pneumonia just after the first cycle of chemotherapy.

## DISCUSSION

Primary CNS-EES, a rare pediatric malignancy that predominantly affects male children aged between 5 and 13 years.^8^ Huguenard et al.^9^ reported occurrence of 40 cases of primary CNS-EES, of which 11 were pediatric patients suffering from both cranial, spinal and combination of both cranial and spinal CNS-EES. This case series presents the clinical details of two male and two female patients diagnosed with CNS-EES, which highlights its occurrence in both genders. This is supported in literature by Cherif et al.^10^ in their systematic review, who reported differences in gender distribution in primary CNS-EES to be non-significant (p>0.05). Similarly, Yang et al.^11^ reported the occurrence of CNS-EES in four children, of which three were females.

The age range of 8 to 13 years, in our case series for patients with primary CNS-EES, aligns with the previously described age distribution of 5 to 13 years in literature^8^. Studies have reported the occurrence of primary CNS-EES on various sites with increased predilection towards cerebral hemispheres, followed by cerebellopontine angles, cavernous sinus, posterior fossa, and spinal cord.^8^,^9^ This is well-explained by the results of our case series in which 50% of patients had a lesion in the cerebral hemisphere, while 25% of patients had pathology in the spinal cord, and 25% had cranial and spinal pathology combined.^8^,^12^

Headache was the most frequently reported presenting complaint of patients, followed by motor deficit, facial palsy, hearing loss, and ataxia. This is also described in literature by Jiang et al.^8^ who reported headache in 50% of patients in his review of case reports followed by motor deficit (30%), facial palsy (30%), hearing loss (20%) and ataxia (10%) in patients suffering from intracranial primary CNS Ewing sarcomas owing to raised intracranial pressure and mass effect while backache and sphincter incontinence in ES originating from cauda equina.^12^

The mean duration of clinical symptoms in our study was 23 days, in contrast to Jiang et al.^8^ who reported a higher mean duration of clinical symptoms, i.e., 5.9 months. This could be attributed to the higher age of patients included in later as literature shows that pediatric ES are detected earlier in comparison to adult patients.^13^ Although consanguinity was positive in 75% of patients in our study, there is no evidence of consanguinity as a risk factor for the occurrence of primary CNS-EES because familial cases for ES have not been reported.^14^ It is proposed to occur due to t (11;22)(q24;q12) translocation, which results in *EWS-FLI1* fusion gene,^8^-^10^ yet the genetic testing was not performed in the majority of our patients due to resource limitations. Almost 50% of patients in our case series had neurocutaneous manifestations in the form of café-au lait spots and pigmented macules, which might indicate underlying Neurofibromatosis (NF) as the upregulation of RAS in NF-1 may act as a mediator of ES indirectly by upregulation of Tyrosine kinases.^15^

Radiologically, these lesions showed heterogeneous contrast enhancement on Magnetic resonance imaging, which is supported in the literature by Jing et al., who reported heterogeneous contrast enhancement in 60% of cases; however, this heterogeneous contrast enhancement might be attributed to the high density of round blue cells in such tumors with excessive protein-rich mucus, hemorrhage, and necrosis.^16^

Histopathological picture of the tumor was similar to the one described previously, with round, small undifferentiated cells with high mitotic activity and hyperchromatic nuclei, suggesting primitive neuroectodermal origin, yet the positive sensitive immunohistochemical markers of ES, i.e., CD99 and NKX2.2, in our case series supported the diagnosis of ES as described in literature.^8^-^10^

The standard treatment protocol for primary CNS-EES is maximal surgical resection along with aggressive chemotherapy and radiotherapy.^8^,^10^ All the patients in our case series underwent maximal safe surgical resection while one of four patients received chemotherapy with the maximum survival of 40 weeks in contrast to four years reported by Jiang et al.^8^ Similarly, the median overall survival in our patients was much less than reported in literature i.e., five weeks in comparison to 13 months as reported in a by Chen et al.^17^ These low overall survival rates observed may be attributed to the fact that only 50% of our patients received chemotherapy within one month after surgery, of which 25% already had metastasis, the poor prognostic marker for primary CNS-EES.^10^ This situation could be linked to the challenges faced in a developing country, owing to delayed diagnosis, longer time period for adjuvant chemotherapy and radiotherapy due to excessive patient turnover in cancer hospitals.

This case series is limited due to small sample size with lost to follow up in 25% of patients, mortality during chemotherapy in 50% leaving only 25% of patients whose chemotherapy is still ongoing, hence the authors are unable to propose effectiveness of regimen, or survival benefits of surgical resection or various chemotherapy options as provided in literature yet this case series provides a valuable insight of primary CNS-EES that should be considered a differential preoperatively when encountering aggressive pediatric CNS tumors. Prospective studies of longer duration and larger cohorts are needed to better delineate the clinical trajectory of this group of patients.

## CONCLUSION

Primary CNS-EES in children is a rare CNS neoplasm, but is not very infrequent in our population. It might have a predilection to parietal lobe involvement with a possible association of cancer predisposition syndromes in populations with high consanguinity. In resource-limited settings with a lack of extensive immunohistochemistry panels and translocation testing, a round blue cell morphology with positive NKX2.2 might serve as a fair substitute for diagnosis, leading to timely, specific treatment.

### Authors’ Contribution:

**RUA:** Concept of study, data acquisition, drafted, and critical review of manuscript.

**SI & RQ:** Data acquisition and drafted the manuscript.

**LUR & MH:** Data interpretation and critically reviewed the manuscript.

**MF:** Data interpretation and critically reviewed the manuscript and Supervision.

All authors have approved the final version of the manuscript to be published and are accountable for the integrity of the study.
